# Supplementary material for: Creating an Implementation Enhancement Plan for a Digital Patient Fall Prevention Platform Using the CFIR-ERIC Approach: A Qualitative Study
Source: Int J Environ Res Public Health. 2023 Feb 21;20(5):3794. doi: 10.3390/ijerph20053794 (PMC10001076; doi:10.3390/ijerph20053794)
Supplement: Supplementary file 1 [file ijerph-20-03794-s001.zip › ijerph-2142908-supplementary.pdf]

Supplementary File One: Intervention Description

Concentric Care Fall Prevention Platform

Introduction

As falls are recognised as multifactorial in aetiology, a range of interventions have been studied to see if they can reduce falls in elderly people. A 2018 Cochrane systematic review aimed to assess the effects of interventions designed to reduce the incidence of falls in older people in care facilities and hospitals[1]. They found there was some evidence that a multipronged prevention approach may reduce falls in hospitals. The multipronged prevention approach could consist of the following components:

- group exercise to improve strength and balance e.g. Tai Chi
- home or hospital-based exercise programs
- osteoporosis treatments
- vitamin D supplementation
- poly pharmacy review
- falls risk screening
- bed and chair sensor alarms
- nurse call systems
- nurse call system induction for patients and residents
- real time location services systems
- falls detection technology

The intelligent design of the care environment can have significant impact on care outcomes, provider and patient experience, and operational and financial performance. The Concentric Care Fall Prevention Platform (hereafter referred to as the Platform) is a smart digital hospital strategy delivered through an enterprise platform that supports end-to-end solutions with simple, standardised, and intelligent workflows.

The Concentric Care Fall Prevention Platform is a digitally-enabled, multipronged prevention approach aimed at reducing falls and reducing harms from undetected falls. The platform augments clinical and lifestyle interventions to reduce patient falls risk.

Platform Components

The Platform brings together a range of technological capabilities focused on delivering clinical outcomes. Each platform capability, outlined below, is designed, and deployed to support clinicians in managing patient falls risk. The strategy and implementation efforts are clinician led and include measurability and data collection as a design requirement.

---

[1] Cameron, I.D., et al., Interventions for preventing falls in older people in care facilities and hospitals. Cochrane Database of Systematic Reviews, 2018(9).

Quiet Environment

A quiet environment is a healing environment and a more pleasant workplace. The elimination of noise pollution and reducing alarm fatigue is a major focus area for smart hospital designers. Research[2] shows that patients heal better in a quiet environment. The Platform quiet environment ensures that alerts that are not clinically critical are delivered discretely and securely to those team members who have a need to know and respond.

Single Device for Clinicians

A key enabler to a smart hospital environment and a quiet environment is the intelligent and rational use of computing devices for clinicians, especially nurses. The Platform includes the selection of a single smart device per clinical role that can serve as the key digital tool for data entry/retrieval, communications, notifications, and location services including duress capability.

There are a wide variety of benefits using a single device strategy, including simplified management complexity and cost, improved staff satisfaction and a more consistent end user experience. When all clinical team members have a smartphone with them, nurse call response times drop dramatically, as immediate voice response to each inquiry is possible regardless of location. The adoption of a single device strategy is an important step in standardising clinical workflows across the enterprise.

The Platform consists of an advanced patient call and communication system which allows patients to call for assistance. It allows the nurse to directly speak back into the room to the patient on their mobile device when the patient calls, reassuring the patient their call has been heard, enabling triage of the call and direction from the nurse to the patient. Patients are less likely to attempt to relocate themselves when they receive a quick voice response from the nursing team.

The Platform has a patient induction video on use of the nurse call system and how its use can help to prevent patient falls. The video can be played on the patient engagement system.

Bed and chair sensor alarms and smart beds can be plugged into the Platform. When a patient is attempting to exit without staff being present, an alarm is raised and sent directly to the mobile device of the primary nurse allocated to the patient. The nurse can directly speak with the patient to provide direction. An alert can also be displayed on the TV annunciators throughout the unit and display on the Journey Board if this is applicable.

Situational Awareness

Situational awareness is an advanced knowledge application of the smart hospital Platform. The Platform aggregates data from systems and sensors via HL7 and FHIR integrations and turns this into useful information. It is the job of a situational awareness application to turn the information from the platform into knowledge that helps care team members perform operational tasks more efficiently.

The Platform uses i) a patient safety dashboard that combines data from an electronic medical record to know which patients are assessed as a falls risk, ii) smart bed API calls to discover the condition of a patient's bed and iii) an in-room privacy preserving 4D radar sensor to detect the

---

[2] Gardner, G., Collins, C., Osborne, S., Henderson, A., & Eastwood, M. (2009). Creating a therapeutic environment: A non-randomised controlled trial of a quiet time intervention for patients in acute care. International Journal of Nursing Studies, 46(6), 778–786. doi:10.1016/j.ijnurstu.2008.12.009

patient's posture and position. This solution sends alerts to nurses on their mobile device as well as the corridor lights and TV annunciators to ensure an immediate response. The Platform can detect an imminent patient fall providing sufficient time to intervene or actual patient fall allowing an immediate care response. The Platform also has manual situational alerts that can be set by clinicians for patients that are assessed as being a falls risk.

In the unfortunate event that a patient fall occurs, the Platform can detect this and raise a critical alarm to the care team. Immediate response to a patient fall is important in minimising the harm experienced from the incident.

Intelligent Messaging Engine

A core component of the Platform is an intelligent messaging engine that powers many of the end-to-end solutions using multi-mode messaging, combining text, voice (one to one and group) and paging. The enterprise messaging engine is centralised and highly available and is integrated to other enterprise systems.

Intelligent messaging facilitates three conceptual classes of communication:

- Person-to-person messaging
- Person-to-system messaging
- System-to-system messaging

Using these three classes of messages, the engine applies contextual heuristics, and increasingly artificial intelligence, to deliver the right message to the right place using the right messaging medium. Typical heuristic inputs are time, clinical criticality, staffing roster and personal preferences. Intelligent messaging engines can eliminate the need for manual calls to coordinate care. This is an important tool for closing the care loop.

Workflow Design

Another key care team coordination topic is end-to-end workflow design. The Platform is designed to simplify, standardise, and automate workflows for improved clinical outcomes. Below is an illustrative example of a smart coordination workflow design for a Code Blue workflow that:

- Is initiated by a physical button press on the nurse call system.
- Leverages an intelligent messaging engine to identify and alert the correct response team.
- Utilises a voice communication system to place all response team members into a conference bridge to consult on the emergency response whilst assembling.
- An enterprise nurse call system that guides the response team to the correct location using 'runway' wayfinding lighting.
- Automatic takeover of the in-room patient engagement devices with a cognitive aide software solution that guides the crash team through best practice resuscitation whilst capturing a detailed record of all crucial data related to the event (Advanced Life Support)
- With an operator console and analytics engine that gives leadership real-time visibility into the event and retrospective reporting for service improvement.

The real time communications of the Platform between care team members using the single device strategy enables all care team members to be constantly reachable by voice or text and able to redirect notifications to other care team members to ensure continuity of service delivery.

Staff Reminders

The Platform allows clinical staff to set them themselves alerts to round or check in with patients at risk. When set the wireless device of the primary nurse receives the alert reminding them the task is due.

Data & Insights

Another essential benefit of the Platform is the ability to standardise data collection across the care delivery continuum to drive continuous improvement and gather predictive analytics. As features are added to the Platform, the data produced from the systems feeds into an analytics engine, which is designed as part of the end-to-end solution. General purpose data warehouses are a useful tool, but they are no substitute for analytics integrated into the end-to-end solution as part of the initial design. Integrated analytics focuses on providing the right context at the right time to the right person which leads to timely intervention to improve the operation.

For continuous improvement initiatives, the platform aggregates patient demographics and care data from the electronic medical record, bed data from the smart bed system, response data from the real-time location and nurse call systems and incidents from the incident tracking system to provide insights into which set of conditions are the most impactful.

The data and insights from the Platform enable the healthcare organisation to take a data-first approach to operational improvement. This approach aligns well with the concept of 'evidence based' medicine. A well-designed smart hospital platform brings the same empirical rigor to hospital operations.

The Platform captures data from several systems into a single repository so that any auditing, compliance, and operational insight can be gained from one place. An appropriate data governance structure is in place to protect privacy and mitigate data theft concerns. Data archival practices are well established to manage cost.

For more information, contact Rauland Australia www.rauland.com.au

Supplementary File Two: Interview Guide

The following questions were used as a guide for the focus groups/interview. Not all questions were asked of participants if they had already provided relevant information to avoid repetition or were potentially interruptive of the flow of conversation. A second interviewer was able to ask any questions that may have been missed or viewed as needing further clarification after the first interviewer completed their questioning.

Background questions

Can you briefly tell us your background (role in this hospital, years of experience)?
What is your experience of using of the Responder Enterprise System?
Can you briefly describe the current falls prevention workflow delivered through Responder Enterprise System?

Key questions

Topic 1: Implementation status

Characteristics of individuals

- At what stage of implementation is the Responder Enterprise System at here at HNELHD New Maitland Hospital?
  - How do you think the program is going?
  - Why do you say that?

Process

- Can you describe the plan for implementing the Responder Enterprise System?
  - How detailed is (was) the plan? Who knows (knew) about it? Is (was) the plan overly complex? Understandable? Realistic and feasible?
  - What is (was) your role in the planning process?
  - Who is (was) involved in the planning process? What are (were) their roles?
  - Are (were) the appropriate people involved in the planning process? How engaged are (were) they?
  - Do you plan to track the progress of implementation based on your plan?
  - What if you have to modify or revise your plan due to barrier, errors, or mistakes?
- Who are (were) the key individuals to get on board with the Responder Enterprise System?
  - To encourage individuals to use the Responder Enterprise System? To help with implementation?

Topic 2: Implementation - compatibility /integration

Inner setting

- Can you describe how the Responder Enterprise System will be (is being) integrated into current processes?
  - How will (did) it interact or conflict with current (falls prevention) programs or processes?
- How well does the Responder Enterprise System fit with existing work processes and practices in your setting?
  - What are likely issues or complications that may arise?

Intervention characteristics

- What kinds of changes or alterations do you think you will need to make (or have been made) to the Responder Enterprise System so it will work (it works) effectively in your setting?
  - Do you think you will be able to make these changes? Why or why not?

Topic 3: Training and resources

Inner setting

- What kind of training is (was) planned for you? For colleagues?
  - Do you feel the training will (has) prepare(d) you to carry out the roles and responsibilities expected of you? Can you explain?
  - What are the positive aspects of planned training?
  - What is missing?
  - What kind of continued training is planned?
- What kinds of information and materials about the Responder Enterprise System have already been made available to you?
  - Copies of materials?
  - Personal contact?
  - Internal information sharing; e.g., staff meetings?
  - Has it been timely? Relevant? Sufficient?

Intervention characteristics

- What supports, such as online resources, marketing materials, or a toolkit, are available to help you implement and use the Responder Enterprise System?
  - How do you access these materials?

Topic 4: Potential impact and acceptance

Outer setting

- How well do you think the Responder Enterprise System will meet the needs of patients at HNELHD New Maitland Hospital?
  - In what ways will the Responder Enterprise System meet their needs?
- What barriers will patients at HNELHD New Maitland Hospital face to participating in the Responder Enterprise System?

Characteristics of individuals

- Do you think the Responder Enterprise System will be effective in your setting?
  - Why or why not?
- How confident are you that you will be able to successfully implement the Responder Enterprise System?
  - What gives you that level of confidence (or lack of confidence)?

Inner setting

- What is the general level of receptivity at HNELHD New Maitland Hospital to implementing the Responder Enterprise System?
  - Why?

Intervention characteristics

- What do influential stakeholders think of the Responder Enterprise System?
  - What do administrative or other leaders think of the Responder Enterprise System?

Intervention characteristics

- How does the Responder Enterprise System compare to other similar programs in your setting?
  - What advantages does the Responder Enterprise System have compared to existing programs?
  - What disadvantages does the Responder Enterprise System have compared to existing programs?

Inner setting

- To what extent might the implementation take a backseat to other high-priority initiatives going on now?
  - How important do you think it is to implement the Responder Enterprise System compared to the other priorities?
  - How important is it to others, such as your co-workers or leaders, to implement the Responder Enterprise System compared to the other priorities

Supplementary File Three: Table of CFIR constructs and respondent numbers

CFIR DOMAIN	Facilitator (1 = yes, 0 = no)	Sum of participants	Barrier (1 = yes, 0 = no)	Sum of participants
Access to Knowledge & Information	1	11	1	11
Adaptability	0	0	1	7
Available Resources	1	4	1	8
Champions	1	3	0	0
Compatibility	1	3	1	8
Complexity	0	0	1	4
Cosmopolitanism	1	5	0	0
Cost	0	0	1	1
Culture	0	0	1	1
Design Quality & Packaging	1	4	1	10
Engaging	0	0	1	1
Evidence Strength & Quality	0	0	0	0
Executing	1	3	1	7
External Change Agents	0	0	1	2
External Policy & Incentives	0	0	1	1
Formally Appointed Internal Implementation Leaders	1	5	0	0
Goals and Feedback	1	2	1	3
Implementation Climate	0	0	1	2
Individual Identification with Organization	1	1	0	0
Individual Stage of Change	1	1	1	6
Intervention Source	0	0	0	0
Knowledge & Beliefs about the Intervention	1	8	1	6

CFIR DOMAIN	Facilitator (1 = yes, 0 = no)	Sum of participants	Barrier (1 = yes, 0 = no)	Sum of participants
Leadership Engagement	1	5	0	0
Learning Climate	1	1	1	1
Networks & Communications	0	0	1	1
Opinion Leaders	1	1	0	0
Organizational Incentives & Rewards	0	0	0	0
Other Personal Attributes	1	1	1	3
Patient Needs & Resources	1	9	1	8
Peer Pressure	1	2	0	0
Planning	1	1	1	6
Readiness for Implementation	0	0	1	1
Reflecting & Evaluating	1	3	1	4
Relative Advantage	1	12	1	2
Relative Priority	0	0	0	0
Self-efficacy	1	5	1	6
Structural Characteristics	1	2	1	4
Tension for Change	0	0	0	0
Trialability	1	1	1	1
